# Can ovarian infertility be treated with bone marrow- or ovary-derived germ cells?

**DOI:** 10.1186/1477-7827-3-36

**Published:** 2005-08-15

**Authors:** Antonin Bukovsky

**Affiliations:** 1 Laboratory of Development, Differentiation and Cancer, Department of Obstetrics and Gynecology, the University of Tennessee Graduate School of Medicine, Tennessee, USA

## Abstract

A year ago, reproductive biologists and general public were astonished with evidence reported by Johnson et al. in Nature 428:145 that mammalian ovaries possess persisting large germline stem cells, which allegedly enable follicular renewal in adult females. Recently, the same research group declared such view obscure, and reported that mammalian oocytes originate from putative germ cells in bone marrow and are distributed by peripheral blood to the ovaries (Cell 122:303). While neglecting available data on the germ cell origin from the ovarian surface epithelium (OSE) in adult mouse and human females and complexity of follicular renewal in humans, the authors widely extrapolated their observations on formation of allogeneic oocytes after bone marrow (or blood) transplantation in ovaries of adult mice treated with cytostatics to clinical implications in the public media. Yet, the resulting outcome that such allogeneic oocytes may enable the propagation of ovarian cycles is a poor alleviation for the women with ovarian infertility. Women lacking primary follicles, or carrying follicles with low quality eggs persisting in aging ovaries, are not concerned about the lack of menstrual cycles or ovarian steroids, but about virtually no chance of having genetically related children. Johnson et al. also reported that the germ cell formation in bone marrow disappears in ovariectomized mice. Such observation, however, raises solid doubts on the bone marrow origin of oocytes. Since germ cells developing from the OSE cells of adult human ovaries during periodical follicular renewal are known to enter blood vessels in order to enable formation of primary follicles at distant ovarian sites, they also contaminate peripheral blood and hence bone marrow. Better knowledge on the complexity of follicular renewal in humans and exploration of a potential of human OSE cells to produce new oocytes in vitro are essential for novel approaches to the autologous treatment of premature ovarian failure and age induced ovarian infertility.

## Comment

The claim that female germ cells in adult mammals (mice and humans) originate from bone marrow and are delivered to the ovaries via the blood stream [1] is another controversial postulate of Johnson et al. Last year this research group, which is lead by Dr. Jonathan L. Tilly of the Massachusetts General Hospital/Harvard Medical School, claimed that large germline stem cells persist in postnatal mammalian ovaries [2]. They allegedly documented that these cells divide and differentiate into new oocytes required for the follicular renewal and concluded: "Therefore, in addition to providing new directions to explore with respect to elucidation the biology of mammalian female germline stem cells, this work has significant clinical implications related to therapeutic expansion of the follicle reserve as a means to postpone normal or premature ovarian failure" [2].

After facing immediate critique, which indicated that such large cells rather resemble earlier described superfluous oocytes leaving mouse and human ovaries [3,4], the Tilly's group now declared that the permanent persistence of female germline stem cells contributing to the follicular renewal in adult mammalian ovaries is obscure [1]. When searching for another original (now extragonadal) source of germ cells, the Tilly's group stated a new surprise for the scientists and general public: Female germ cells cyclically develop in bone marrow of the adult mammalian females [1]. Yet, the experienced reproductive biologists stay calm. Why? There is no need to oppose this new "discovery" for another year or so, since the authors killed their claim instantly, by themselves, and in the same article.

To explore an idea on the extragonadal origin of germ cells in adult mammalian females, one will compare observations in animals with and without ovaries. Johnson et al. did and reported: In the absence of ovaries the evidence of germ cell formation in bone marrow completely disappears. However, instead of realizing that this observation is a perfect evidence on ovarian origin of germ cells, which enter the ovarian blood stream and, therefore, some of them bone marrow too, the authors surprised the reader with an additional uncovering: "The results from the ovariectomy experiments lend further support to the existence of a novel communication loop between the ovaries and bone marrow that may regulate the extent of de novo oocyte production each cycle" [1].

If Johnson et al. are more respectful for the available literature, they will consider that the germ cells derived from ovarian surface epithelium (OSE) utilize blood vessels for colonization of distant targets during follicular renewal [3], and hence contaminate peripheral blood and bone marrow. In other words, instead of the periodical flow of putative bone marrow-derived germ cells to the ovaries, the ovarian germ cells may periodically enter bone marrow via the blood stream.

Curiously, although Johnson et al. are apparently aware of relevant literature, the studies on the germ cell and oocyte origin from OSE and follicular renewal in adult mouse [5,6] and human ovaries [3,7] are unprofessionally ignored. They were neither referenced in the introduction nor discussed as a possible alternative on the origin of germ cells and oocytes in articles of Johnson et al. on persisting germline stem cells [2] or on bone marrow origin of germ cells in adult mammalian ovaries [1]. Although article of Allen from 1923 entitled "Ovogenesis during sexual maturity" [5] was recently mentioned by Johnson et al. [1], the idea on the oocyte origin from OSE in adult mammals has not been acknowledged.

Two highly distinguished American pioneers of modern reproductive physiology in 1920s and 1930s, Edgar Allen and Herbert M. Evans, concluded their studies of oogenesis and follicular renewal in adult mouse ovaries as follows: "A cyclical proliferation of the germinal epithelium (OSE) gives rise to a new addition of young ova to the cortex of the adult ovary at each normal oestrous period" [5] and "New oocytes are formed throughout life, and in phase with the reproductive cycle, from germinal epithelium of the adult mammal, at the same time as vast numbers of already-formed oocytes become eliminated through atresia" [6]. Why these articles are not appropriately referenced by Johnson et al.? The last year "discovery" on follicular renewal in postnatal mouse ovaries, which compensates concomitant atresia [2], has been, in fact, described more than 70 years ago.

What Johnson et al. have recently shown is that the mouse ovaries lacking primary follicles after treatment with cytostatics exhibit follicles with allogeneic oocytes following bone marrow or peripheral blood transplantation from distinct mouse donors. The concomitant immunosuppression may enable allogeneic oocytes to develop, either from the donor-derived circulating ovarian germ cells, or due to the transplantation of immune-system related cells (blood monocytes and lymphocytes), which accompany as ovarian macrophages and T lymphocytes the development of germ cells from some OSE cells in adult [7] and fetal human ovaries [8]. Yet, the resulting outcome that such follicles with allogeneic oocytes "play a critical role in the propagation of each ovarian cycle" [1] represents a poor promise for the women with ovarian infertility. Women lacking primary follicles with own oocytes, or carrying follicles with low quality eggs persisting in aging ovaries, are not concerned about the lack of menstrual cycles or ovarian steroids, but about virtually no chance of conceiving and having genetically related children.

Recently, we suggested animal experiments comparing the effect of autologous vs. allogeneic white (nucleated) blood cell concentrate (buffy coat) transfusions for induction of follicular renewal after chemotherapy in order to determine possible advantage of the former against the latter procedure (submitted May, 2005). Yet, compared to the small laboratory rodents with available clusters of primitive granulosa cells resembling human fetal ovaries [8], a re-colonization of adult human ovaries with new primary follicles requires their readiness by the means of the presence of nests of primitive granulosa cells. Such nests are required for follicular renewal, since superfluous oocytes are not preserved and degenerate [3]. In other words, even transplantation (transfusion) of autologous germ cells may not be sufficient for follicular renewal in aging women, which lack nests of primitive granulosa cells in their ovaries (unpublished observations). On the other hand, one may imagine a collection of autologous blood sample during ovarian oogenesis in younger females prior to anti-cancer chemotherapy or autologous white blood cells, and subsequent (after chemotherapy) rejuvenation of younger ovaries, which may contain nests of primitive granulosa cells ready to form primary follicles.

However, the age limitless *in vitro *production of new autologous eggs from the OSE cells of human ovaries [8-10], and their *in vitro *fertilization and utilization of embryos for intrauterine implantation, may represent more suitable variant for providing genetically related children to women with ovarian infertility, worth of consideration and further exploration.
